# Muscle Activity Onset Prior to Landing in Patients after Anterior Cruciate Ligament Injury: A Systematic Review and Meta-Analysis

**DOI:** 10.1371/journal.pone.0155277

**Published:** 2016-05-11

**Authors:** Daniel Theisen, Isabel Rada, Amélie Brau, Paul Gette, Romain Seil

**Affiliations:** 1 Sports Medicine Research Laboratory, Luxembourg Institute of Health, Strassen, Luxembourg, G.-D. of Luxembourg; 2 Exercise Science Laboratory, Faculty of Medicine, Universidad Finis Terrae, Santiago, Chile; 3 Department of Orthopedics, Centre Hospitalier de Luxembourg, Strassen, Luxembourg, G.-D. of Luxembourg; Bern University of Applied Sciences, SWITZERLAND

## Abstract

Muscle activation during landing is paramount to stabilise lower limb joints and avoid abnormal movement patterns. Delayed muscle activity onset measured by electromyography (EMG) has been suggested to be associated with anterior cruciate ligament (ACL) injury. Therefore, the aim of this systematic review and meta-analysis was to test the hypothesis if ACL-injured patients display different results for muscle onset timing during standard deceleration tasks compared to healthy control participants. PubMed, Embase, Scopus and ScienceDirect databases were systematically searched over the period from January 1980 to February 2015, yielding a total of 1461 citations. Six studies meeting inclusion criteria underwent quality assessment, data extraction and re-computing procedures for the meta-analysis. The quality was rated “moderate” for 2 studies and “poor” for 4. Patients included and procedures used were highly heterogeneous. The tasks investigated were single leg hopping, decelerating from running or walking, tested on a total of 102 ACL-injured participants and 86 controls. EMG analyses of the muscles vastus lateralis, vastus medialis, lateral and medial hamstrings revealed trivial and non-significant standardised mean differences (SMD<0.20; p>0.05) between patients and control participants. Furthermore, no differences were found between the contralateral leg of patients and controls for muscle activity onset of the medial and lateral gastrocnemius (SMD<0.20; p>0.05). Based on 3 studies, the involved legs of ACL-injured patients showed overall earlier muscle activity onset compared to control participants for the medial gastrocnemius (SMD = 0.5; p = 0.05). Similar results were found for the lateral gastrocnemius (SMD = 2.1; p<0.001), with a greater effect size but based only on a single study. We conclude that there are no differences between leg muscles of ACL-injured patients and healthy controls regarding the muscle activity onset during landing. However, current evidence is scarce and weak, which highlights the need for further research in this area.

## Introduction

Non-contact anterior cruciate ligament (ACL) injuries commonly occur in a sport context, during abrupt deceleration movements associated with sudden changes of direction, such as cutting, pivoting or landing movements. These manoeuvres challenge balance control and can lead to abnormal loading and injury of the ACL [1]. Most non-contact ACL ruptures occur with a knee angle of less than 30 degrees and a malalignment of the ankle, knee and hip joints [2, 3], resulting in the so-called valgus collapse [4]. ACL injury causes acute loss of function and is often associated with prolonged absence from the sports field, early athlete retirement and a high incidence of osteoarthritis development [5]. Furthermore, the literature reports a 15 times greater risk of sustaining a second injury, with a tendency towards higher rates in the contralateral than in the ipsilateral leg [6]. The restoration of function to a comparable level as prior to injury depends critically on the rehabilitation process and requires a strong focus on muscle strength, balance training and proprioception exercises [7, 8].

Standardized questionnaires, thigh circumference and laxity measurements, as well as isokinetic testing and dynamic function tests are generally used to evaluate if the patient is ready to return to sport [9]. Athletes who do not meet minimal return-to-sport criteria may lack neuromuscular control and develop impaired movement patterns. Neuromuscular control has been defined as *“an unconscious activation of dynamic restraints*, *occurring in preparation for and in response to joint motion and loading*, *for the purpose of maintaining and restoring functional joint stability”*[10]. Deficient neuromuscular control can generate increased loads on the knee joint during activities of daily living and produce cartilage damage over time [11]. On the other hand, abnormal movement patterns observed post-ACL injury could represent a protective mechanism to avoid excessive shear forces at the knee joint in ACL-deficient or -reconstructed individuals [12]. In the latter case, mechanical stabilization of the knee joint has been restored, but neurosensory deficits may still persist, an aspect that needs evaluating [13].

Surface electromyography (EMG) is a technique that has been widely used to evaluate neuromuscular function and muscle recruitment patterns during standardised landing tasks [14, 15] with acceptable reliability [16]. Appropriate muscle activation prior to ground contact increases the sensitivity of the muscle spindles and could contribute to proper joint stiffness and stabilisation during landing [17]. The onset timing of muscular activity is therefore a particularly interesting variable to describe control strategies during deceleration tasks. Muscle activity onset timing can be evaluated via EMG by detecting the first motor units action potentials that build up constantly before touchdown [15, 18].

A recent case study suggests that delayed muscle activity onset may be a risk factor for ACL injury [19]. Onset timing has previously been investigated as an indicator of muscular activation to compare ACL injured individuals with healthy controls. Indeed, this factor could be an important aspect in the risk profile definition of a patient or the return-to-play decision. Although some differences have been suggested between ACL patients and healthy individuals, the existing evidence is scarce and unclear. We therefore aimed to summarize the current scientific knowledge by conducting a systematic review and meta-analysis of the available literature on the muscle onset timing prior to ground contact during deceleration tasks. We hypothesized that ACL-injured patients would display significantly different results for muscle onset timing compared to healthy control participants. Between group differences were investigated for the ACL injured limb as well as for the healthy contralateral leg of the patient group.

## Methods

This systematic review followed the guidelines and explanations of the Preferred Reporting of Systematic Reviews and Meta-analyses (PRISMA) [20] statement (S2 Table). No protocol for this systematic review was pre-registered.

### Search strategy

The following databases were searched: PubMed, Embase, Scopus and ScienceDirect, from the beginning of January until the middle of February 2015. The following strategy with four keywords was used: (“Anterior Cruciate Ligament” OR “ACL”) AND (“Electromyogra*” OR “EMG”). The search was restricted to publications from 1980 to February 2015 in French, German and English.

### Eligibility criteria and study selection

After removing the duplicates, two authors (AB and IR) reviewed all the titles and abstracts to determine their possible eligibility for the review. The inclusion criteria were as follows: 1) type of study—randomized control trials, cross-sectional studies and cohort studies; 2) type of participants—ACL-deficient or -reconstructed patients, being compared to their healthy contralateral leg or to injury-free control participants, with a minimum age of 18 years and regardless of their physical activity level; 3) type of measurement—EMG activity of lower limb muscles prior to landing from a deceleration task, defined as the terminal swing/areal phase in actions such as walking, running, cutting, pivoting manoeuvres or landing from a jump; 4) type of outcomes—detection of muscle activity onset time, defined as the first record of motor unit action potentials [15] and determined by a clearly described detection technique. Excluded articles were discussed by both authors (AB and IR) and any disagreement was resolved by consensus.

Full text versions of the studies considered eligible after abstract screening were independently assessed by both authors (AB and IR) to confirm the inclusion. The examiners reviewed the selected full text articles, classified them and sorted them by the type of deceleration task, the reporting of EMG data before touchdown, recorded EMG variables (onset/amplitude) and participants compared (uninjured contralateral leg or healthy control group). In addition, the units of measurement were identified (values reported in milliseconds [21], as percentage of stance phase [%SP] or gait cycle time [%GCT]) and evaluated for possible data extraction and conversion into ms. Studies with amplitude measurements only, as well as sole graphic representations of onset data, were excluded by consensus of the 3 reviewers (AB, IR and PG) after a detailed full text examination. The studies were included in the meta-analysis if average onset values and corresponding dispersion measures were reported, i.e. standard deviation (SD) or standard error (SE).

### Quality assessment

To assess the quality of the studies, the checklist of Munn et al. [22], derived from the Downs et al. check list [23] was selected and further adapted for the specific requirements of this systematic review. Details are provided in the complete item list of the quality assessment, which is available as supporting information file S1 Table. Four new items were added for the quality assessment of the EMG methodology applied, replacing item 20 of Munn’s list. These new items were based on the current “Standards for reporting EMG data”, which are constantly updated and presented by the Journal of Electromyography and Kinesiology [24]: (1) Information about electrode type and placement (electrode type, electrode location, skin preparation, inter-electrode distance, and crosstalk testing), (2) Instruments used for the EMG signal recording (software/hardware and sampling frequency used), (3) EMG data treatment (filter types used, selected cut-off frequencies and described onset detection methodology), (4) efforts of time synchronization for all recorded signals. Regarding these four new items, each question was answered “Yes” if the item was clearly described (score = 1), or “No” if the information was unclear, incomplete or not reported (score = 0).

Further adjustments in the check list were performed to assess the risk of bias. The original items 11 and 12 of the Munn list, describing the individuals approached for participation and those prepared to be tested, were merged into a single new item (item 8). Also, the original item 15 of Munn’s list relative to blinding was supplemented and given a new number (item 9) to assess if the examiner was blinded in the case where visual inspection of the EMG data was used to measure muscle activity onset, either as the only method or as a confirmation of the results provided by an algorithm. The original item 16 was omitted. The original item 18 (new item 10) was complemented with the arguments of Weissgerber et al. [25] who stated that a parametric statistical test is appropriate if symmetric distribution is confirmed. The original items 20 and 21 of Munn’s list were omitted and replaced with the new item 15, evaluating the matching of the control group and its statistical evidence. Finally, the original item 25, assessing the reporting of separate results in case of an inhomogeneous distribution (sex, age, activity level) of participants or the presence of participants with both conservative and surgical treatment was numbered item 16. The complete item checklist applied for the present study, including comments and references, can be found in supporting information file S1 Table.

Two reviewers (AB and IR) scored each selected study independently. The outcomes were discussed together with PG and DT to set the final scores for each paper, any disagreement being solved by consensus. Once the items were scored, the number of questions with a positive response was computed and expressed in percentage. We used the Munn’s list [22] scoring scale to determine the quality of each study according to their percentage reached: high for a score over 75%, moderate for a score between 60–74% and low for a score below 60%.

### Data management

The following information was extracted independently by two authors (AB and IR) from the selected articles: authors and year of publication, total and sub-group participant number (according to sex, age, type of treatment and available data for group comparisons concerning the ACL-injured or the contralateral legs and the dominant or non-dominant control legs or their average), task performed, muscles tested, onset determination method (visual inspection or the mathematical algorithm used) and data units (ms, %SP or %GCT). Furthermore, average values for activity onset and the corresponding SD or SE of the following muscles were extracted: medial hamstrings, lateral hamstrings (biceps femoris), vastus lateralis, vastus medialis, gastrocnemius medialis and gastrocnemius lateralis. The EMG results used for the medial hamstrings were gathered either from the semitendinosus muscle [26–28] or the semimembranosus muscle [29], given their similar function and the high degree of cross-talk in their surface EMG signals [30]. For those studies focusing on walking, only results obtained during horizontal walking and at self-selected speed were used.

### Data handling of specific studies

Due to variabilities within the studies in the data reported, additional calculation steps were carried out for the meta-analysis. When SE was presented in a study as the dispersion measure, the latter was transformed into SD according to the Cochrane Handbook for Systematic Reviews of Interventions [31].

For the control groups, some studies provided only results from one leg (i.e. dominant or non-dominant). However, when the studies provided values from both legs, we calculated the pooled mean and standard deviation for each muscle based on the equation proposed by Srivastava et al. [32]. This decision was based on a recent literature review that did not find any significant differences between both limbs during functional tasks in healthy individuals [33]. In the case of studies comparing different types of patients (i.e. ACL-deficient and -reconstructed) to the same control group, patient data were pooled to avoid including the same control group twice in the meta-analysis [31].

### Statistical methods

Due to the heterogeneity of the data among the studies, a random effects meta-analysis was conducted on the results from the muscles investigated (vastii, hamstrings and gastrocnemius) and reported separately by tasks investigated (jumping, deceleration from running and walking). Since the studies had different types of data, the individual scales were aligned to point in the same direction and the standardized mean difference (SMD) was used to adjust the results to a uniform scale, [31]. The extracted means and SDs were used to calculate the SMD and the corresponding 95% confidence intervals (95% CIs). All statistics were performed using the Cochrane review manager software (Review Manager (RevMan) [Computer program]. Version 5.3. Copenhagen: The Nordic Cochrane Centre, the Cochrane Collaboration, 2014). The SMD was interpreted as trivial <0.20, small 0.20–0.59, moderate 0.60–1.19 and large ≥ 1.2 according to the Cohen’s modified scale [34]. Negative SMD values indicate that the muscles of ACL-deficient or -reconstructed patients have lower onset values compared to the control group, thus reflecting later onset of muscle activity prior to contact with the ground (i.e. shorter activity onset time in patients). The group difference was statistically significant when the 95% CIs for the SMD did not contain zero. Heterogeneity was assessed by the *I²* value [35] and classified as not important for values between 0% and 40%, moderate heterogeneity for values between 30% and 60%, substantial heterogeneity for values between 50% and 90% and considerable heterogeneity for values between 75% and 100% [31].

## Results

### Search results

**Fig 1:**
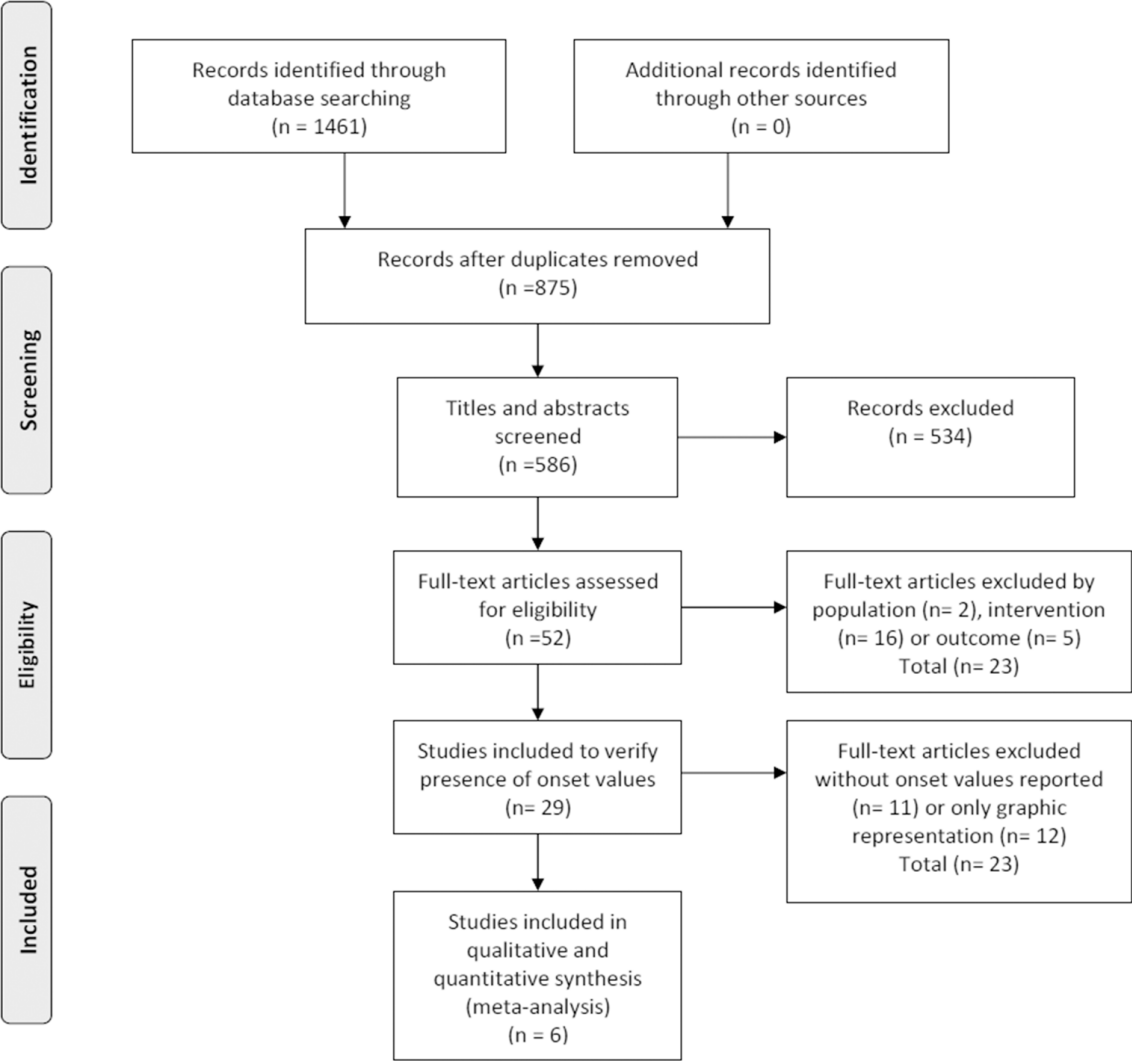
Search results throughout the review process.

The search made in the databases yielded 1461 citations comprising 875 duplicates that were removed (Fig 1). By screening titles and abstracts, another 534 were excluded since they did not met the set inclusion criteria: 185 by population, 201 by intervention (no EMG measurement during deceleration task before ground contact), 14 by comparative group (healthy participants or contralateral uninjured leg missing), 14 by outcome (no statistical EMG analysis of the deceleration task reported), 83 by study design, 25 by language and 12 by animals. The full texts of the remaining 52 studies were screened and 23 were excluded (16 by intervention, 5 by outcome and 2 by population). The 29 studies left, were reclassified and 23 of them excluded, 11 because they did not report proper onset values, and 12 because outcomes were reported by graphical representation only. The remaining six studies [26–29, 36, 37] met all of the eligibility criteria and were grouped according to their task investigated (i.e. jumping, running, walking).

### Quality assessment of included studies

The quality rating of the included studies revealed scores between 28% and 72%, with 2 studies having moderate and the remaining 4 low quality scores (see supporting information file S3 Table). Only 2 trials presented a clear description of their hypothesis/aim/objective. However, all of them described the main outcomes and the onset measuring method used. The inclusion/exclusion criteria and the participant recruitment procedure were found to be described at the same time in one study. The distribution of potential confounders like sex, age and type of treatment was clearly reported in 4 studies. The main findings were sufficiently described in 3 papers, and all 6 studies provided some random variability estimates (i.e. SD, SE).

Only one study indicated the source of ACL patients, described their selection and the proportion of the population from which they were derived. In 4 studies some sort of blinding was achieved, either through the use of an algorithm to determine muscle activity onset, or through blinding of the assessor with regards to the participant group being analysed. The statistical tests applied were found to be appropriate in 5 trials. Regarding the newly developed item list concerning the EMG methodology used to determine onset times of muscular activity (items 11 to 14), all studies failed to fully describe the electrode type and placement protocols. The instruments used for EMG testing were specified in 3 articles only. Furthermore, the data treatment was reported in 5, and the signal time synchronization in 4 studies. Finally, the checklist revealed that no study presented statistical evidence to confirm the absence of significant differences between the distributions of ACL-injured participants and controls. Some authors reported that populations of participants were matched but did not provide any statistical results. However, two studies presented separate results in subgroups, showing in these cases that adjustments for one of the main confounders (sex or type of treatment) were applied.

### Characteristics of included studies

#### Methods

**Fig 2:**
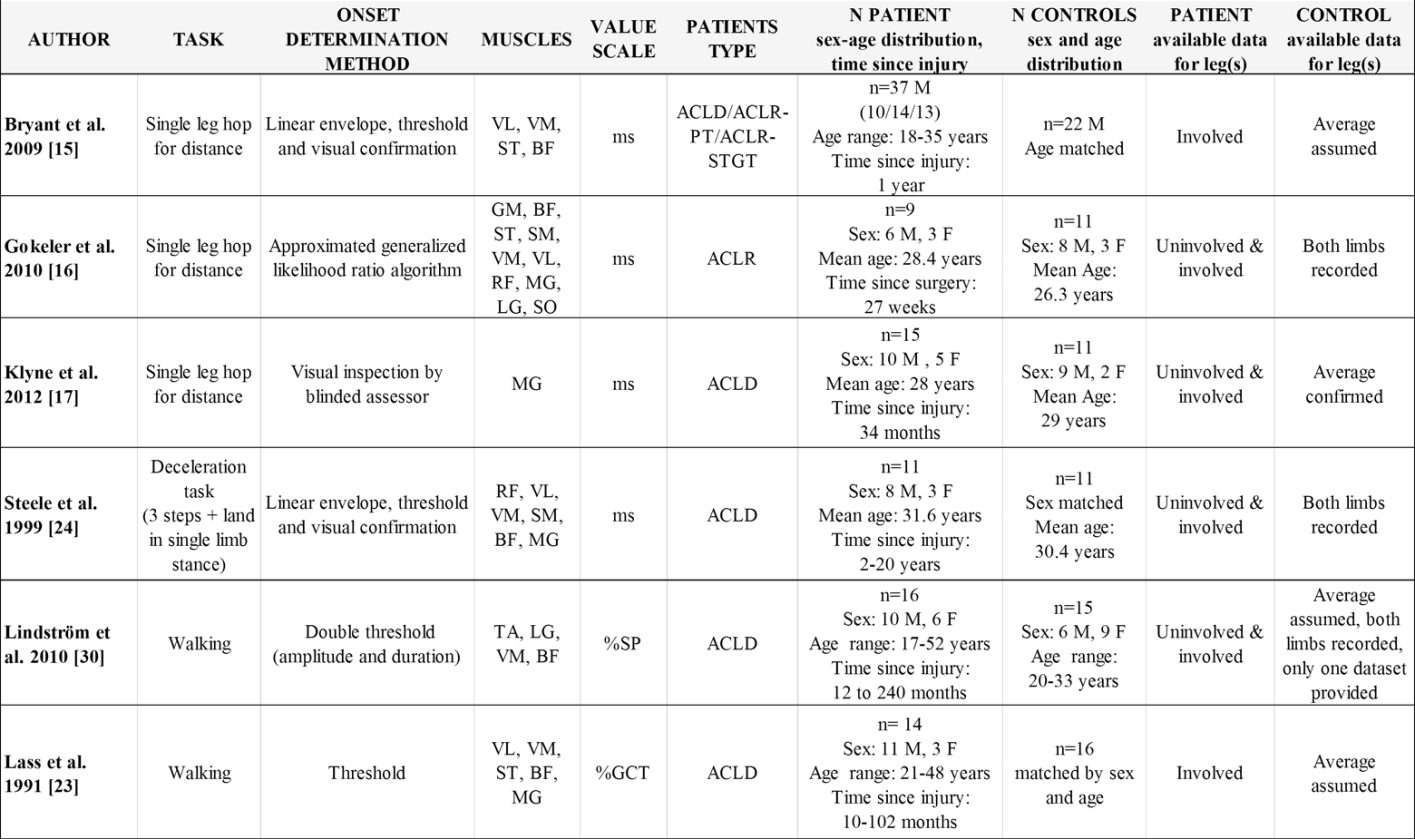
Overview of the main studies characteristics. ACLD, ACL-deficient; ACLR, ACL-reconstructed; PT (patellar tendon graft); STGT (semitendinosus-gracilis tendon graft); ms, milliseconds; %SP, percentage of stance phase; %GCT, percentage of gait cycle time; VL, vastus lateralis; VM, vastus medialis; RF, rectus femoris; ST, semitendinosus; SM, semimembranosus; BF, biceps femoris; GM, gluteus maximus; MG, medial gastrocnemius; LG, lateral gastrocnemius; SO, soleus; TA, tibialis anterior. Note that in the study of Lindstrom et al, the final number of included participants is lower due to technical issues related to the EMG recordings.

The main characteristics of the six selected studies are presented in (Fig 2). All were case-control trials published in English. Four of them reported values for the patients’ injured leg, the healthy contralateral leg and the healthy control group. Two studies reported the results of the injured leg and the control group. Regarding the task performed, three studies investigated the single leg hop for distance. Bryant et al. [26] allowed a counter movement prior to the hop and asked the participants to jump as far as possible during five barefoot trials. Gokeler et al. [27] allowed the participants to use their preferred technique for the same task, wearing their own sport shoes during 3 trials. The participants of the study of Klyne et al. [36] were instructed to jump barefoot over a predetermined distance. The second type of task included in this review was proposed by Steele et al. [29]. It comprised three quick steps to catch a ball at chest level, followed by a single leg landing while being shod. Finally, walking represented the third type of task investigated, but still with considerable between-study differences. The trial of Lindstrom et al. [37] described a barefoot walk with a self-selected speed on a 10 meter run way, while Lass et al. [28] tested their participants with pre-selected speed at different treadmill gradients (0%, 5%, 10%, 15%, 20% and 25%) while being shod.

#### Participants

The included studies involved 102 ACL injured participants (51 with values of their contralateral leg) and 86 controls, with an age ranging between 17 to 52 years. Three studies were performed with participants from Australia, one from Denmark, one from The Netherlands and another one from Sweden. In patients, diagnosis of ACL injury was confirmed arthroscopically in 3 studies [26, 28, 37]. The time since injury to the testing varied within the trials, from 10 months to 20 years for ACL deficient and from 27 to 48 weeks after surgery for ACL reconstructed patients.

#### Secondary outcomes

Beside the muscle activity onset measurements prior to initial contact with the ground, other variables were considered. Some studies implemented questionnaires like the Cincinnati, IKDC, KOOS and Lysholm score. Knee laxity was measured in 4 studies, including also other kinetic and kinematic variables such as tibial acceleration, joint angles, ground reaction forces and tibio-femoral shear forces.

### ACL injured leg of patients vs control participants

**Fig 3:**
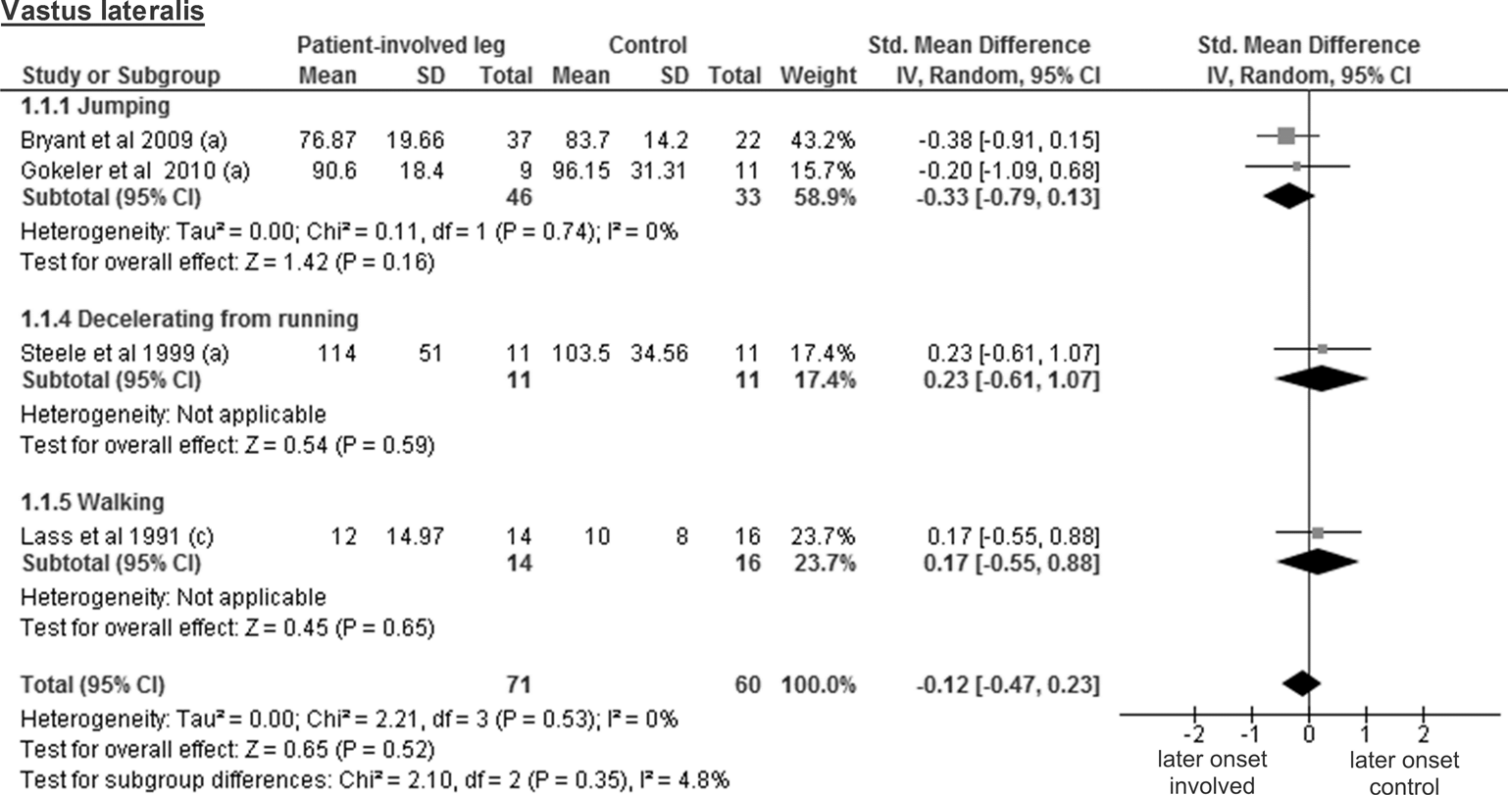
Forest plot illustrating muscle activity onset times of both groups and group differences for vastus lateralis. SMD between ACL patients’ involved leg (Patient-involved leg) and control participants (Control). (a), values in milliseconds; (c), values in percentage of gait cycle time.

**Fig 4:**
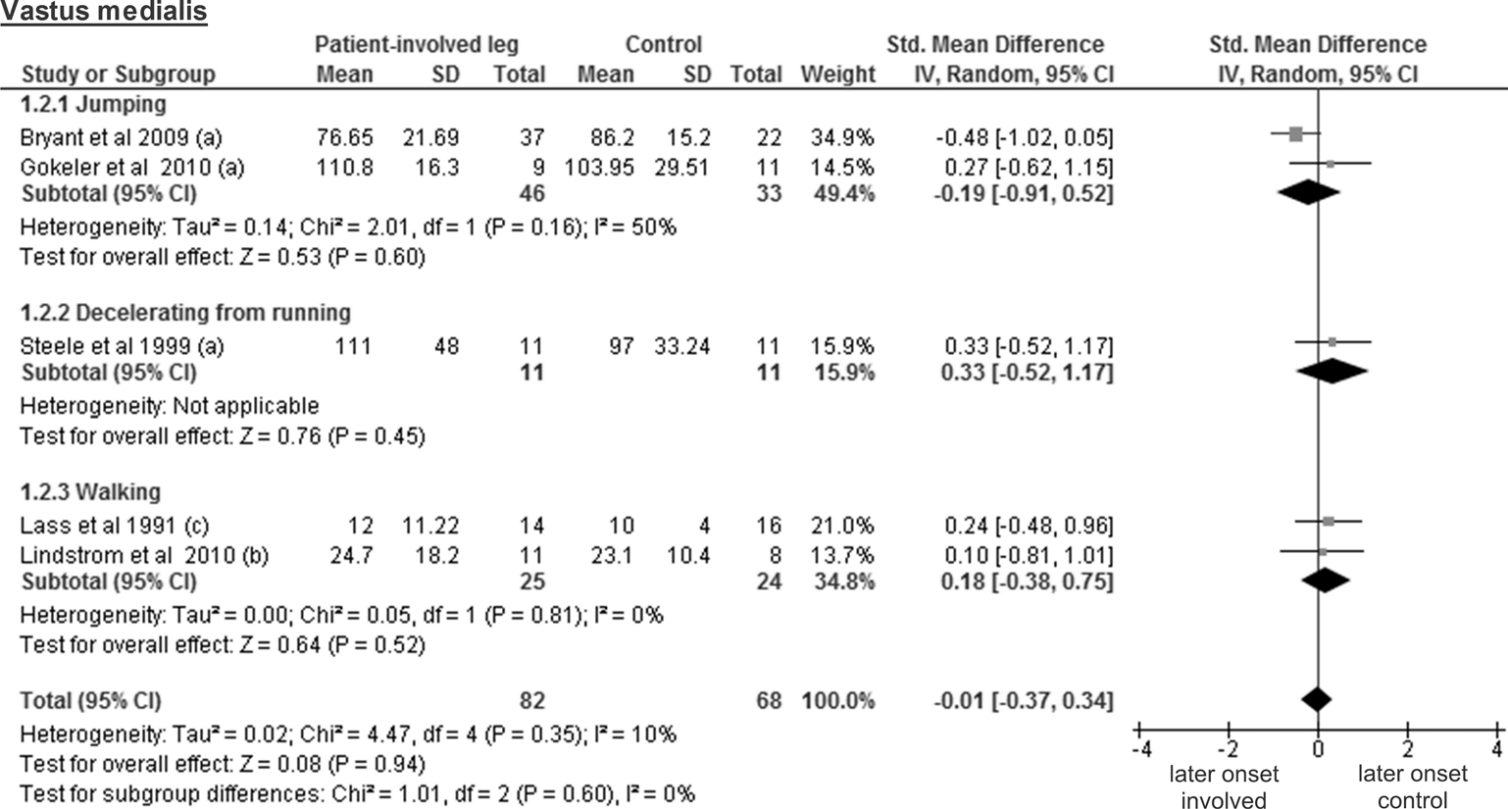
Forest plot illustrating muscle activity onset times of both groups and group differences for vastus medialis. SMD between ACL patients’ involved leg (Patient-involved leg) and control participants (Control). (a), values in milliseconds; (b), values in percentage of stance phase; (c), values in percentage of gait cycle time.

As already mentioned above, patient and control participants were compared using SMD, with negative values indicating shorter activity onset time in patients. The available data of the vastus lateralis activity onset from four studies including 71 patients and 60 controls revealed a trivial and non-significant SMD of -0.12 (95% CI -0.47 to 0.23; p = 0.52) (Fig 3). The results regarding the vastus medialis muscle stem from five studies on 82 patients and 68 controls. Again, a trivial and non-significant SMD of -0.01 was found (95% CI -0.37 to 0.34; p = 0.94) (Fig 4).

**Fig 5:**
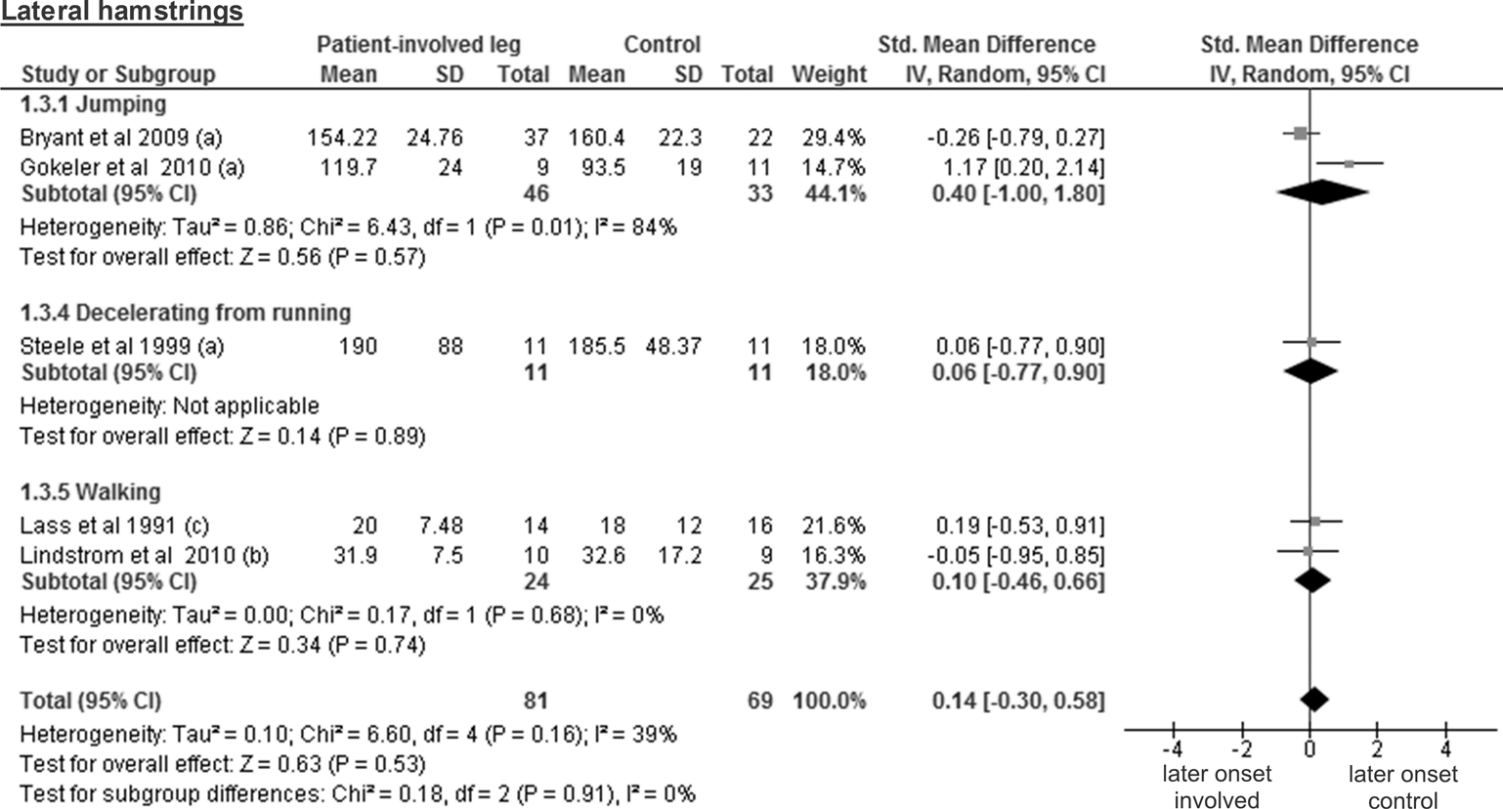
Forest plot illustrating muscle activity onset times of both groups and group differences for lateral hamstrings. SMD between ACL patients’ involved leg (Patient-involved leg) and control participants (Control). (a), values in milliseconds; (b), values in percentage of stance phase; (c), values in percentage of gait cycle time.

**Fig 6:**
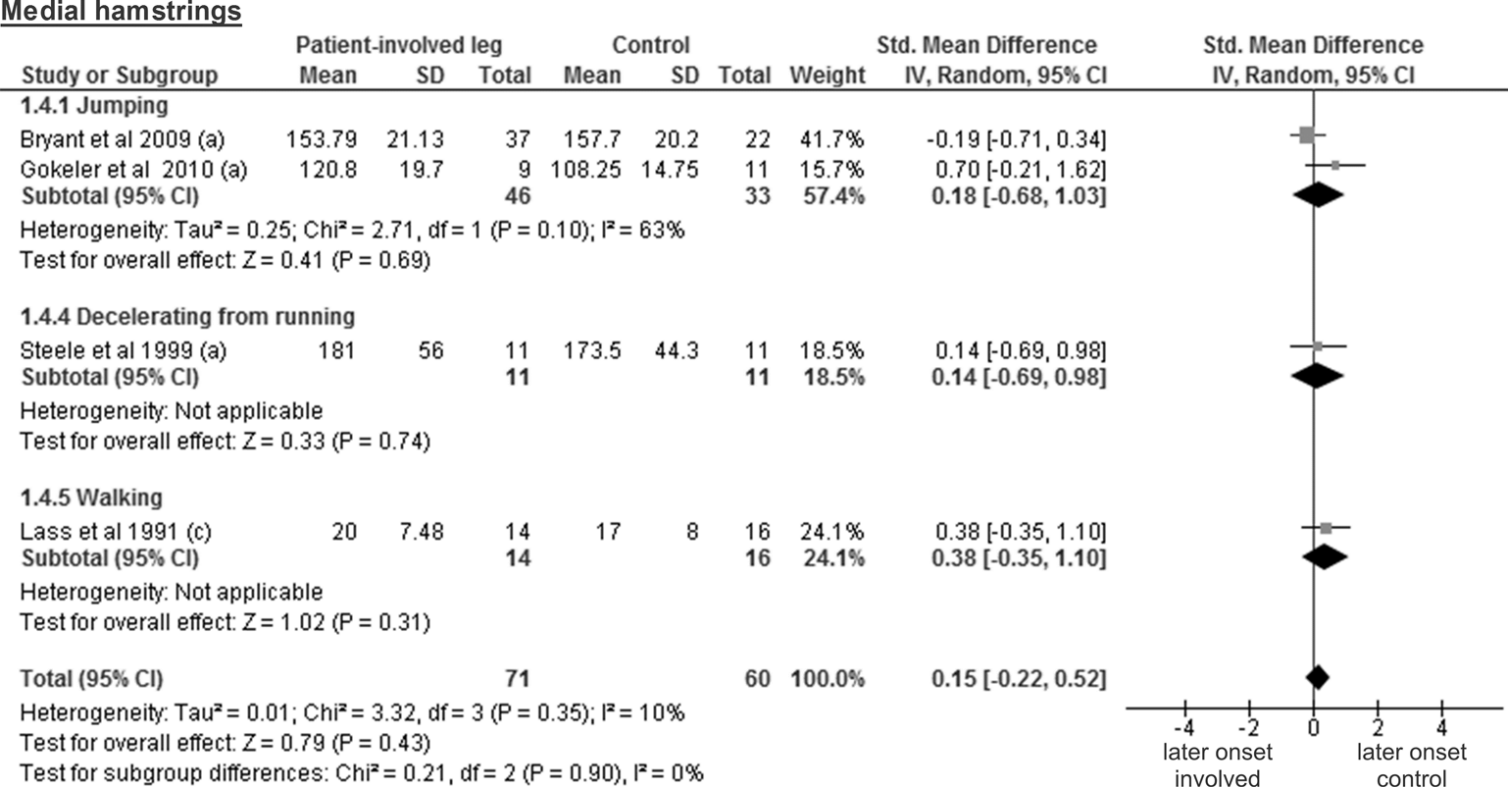
Forest plot illustrating muscle activity onset times of both groups and group differences for medial hamstrings. SMD between ACL patients’ involved leg (Patient-involved leg) and control participants (Control). (a), values in milliseconds; (c), values in percentage of gait cycle time.

The analysis of five studies that tested lateral hamstrings from 81 patients and 69 controls revealed a trivial and non-significant SMD of 0.14 (95% CI -0.30 to 0.58; p = 0.53) (Fig 5). The medial hamstrings were assessed in four studies including 71 patients and 60 controls, resulting also in a trivial and non-significant SMD of 0.15 (95% CI -0.22 to 0.52; p = 0.43) (Fig 6).

**Fig 7:**
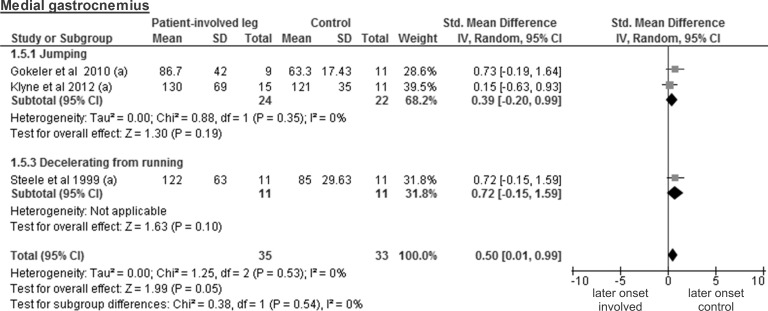
Forest plot illustrating muscle activity onset times of both groups and group differences for medial gastrocnemius. SMD between ACL patients’ involved leg (Patient-involved leg) and control participants (Control). (a), values in milliseconds.

Finally, the analysis of three studies on 35 patients and 33 controls for the medial gastrocnemius showed a small but significant SMD of 0.50 (95% CI 0.01 to 0.99; p = 0.05) (Fig 7). The calculation for the lateral gastrocnemius was based on a single study [27] that tested 9 patients and 11 control legs and revealed a large and significant SMD of 2.13 (95% CI 0.98 to 3.28; p<0.001).

### Healthy contralateral leg of ACL patients vs control participants

**Fig 8:**
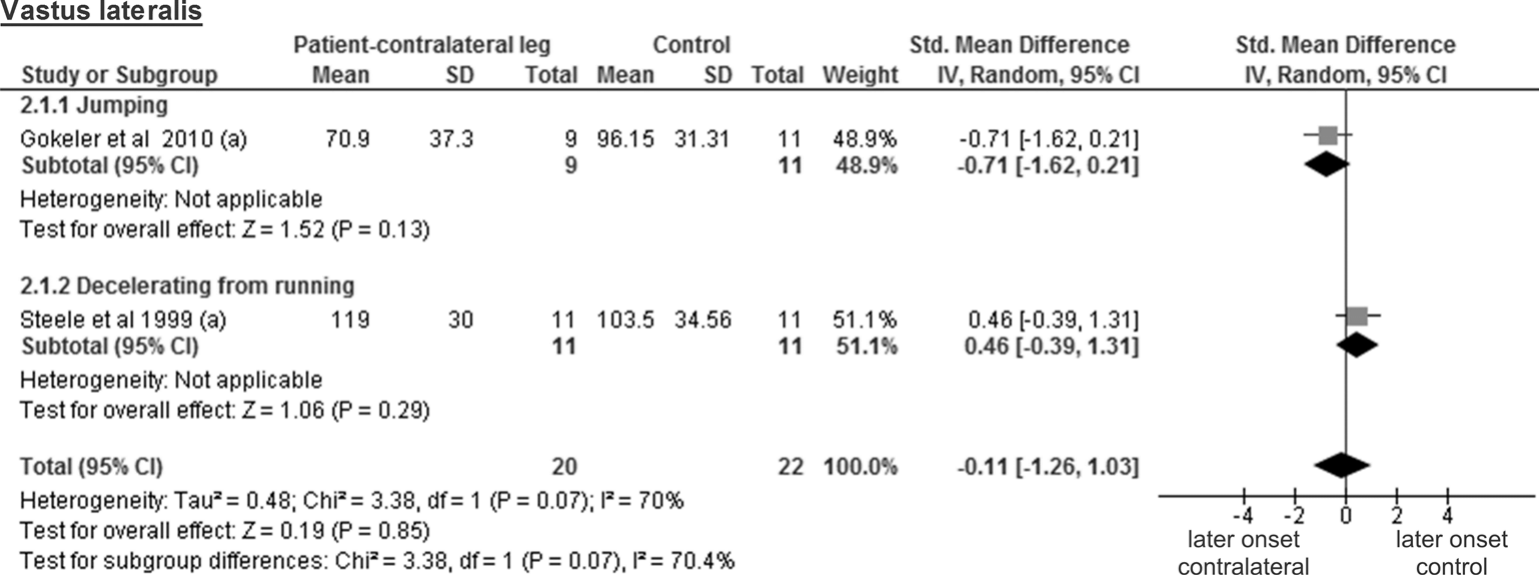
Forest plot illustrating muscle activity onset times of both groups and group differences for vastus lateralis. SMD between ACL patients’ healthy contralateral leg (Patient-contralateral leg) and control participants (Control). (a), values in milliseconds.

**Fig 9:**
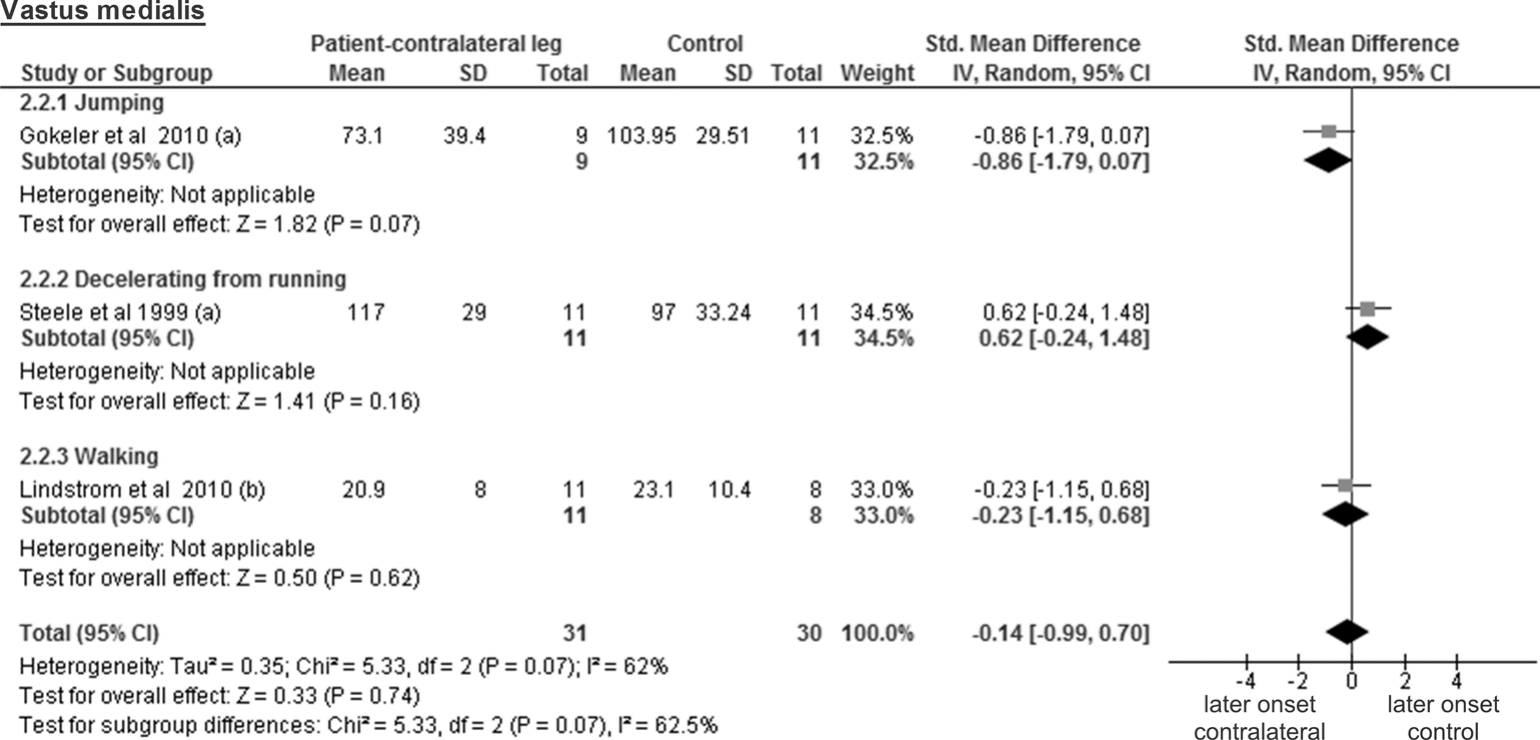
Forest plot illustrating muscle activity onset times of both groups and group differences for vastus medialis. SMD between ACL patients’ healthy contralateral leg (Patient-contralateral leg) and control participants (Control). (a), values in milliseconds; (b) values in percentage of stance phase.

The comparison between the results from healthy contralateral legs of 20 patients and those from 22 controls for the vastus lateralis muscle stems from 2 studies and showed a trivial and non-significant SMD of -0.11 (95% CI -1.26 to 1.03; p = 0.85; considerable heterogeneity of I² = 70%) (Fig 8). Three studies tested the vastus medialis in a total of 31 healthy contralateral patient legs and 30 legs of control participants, revealing a trivial and non-significant SMD of -0.14 (95% CI -0.99 to 0.70; p = 0.74; considerable heterogeneity of I² = 62% (Fig 9).

**Fig 10:**
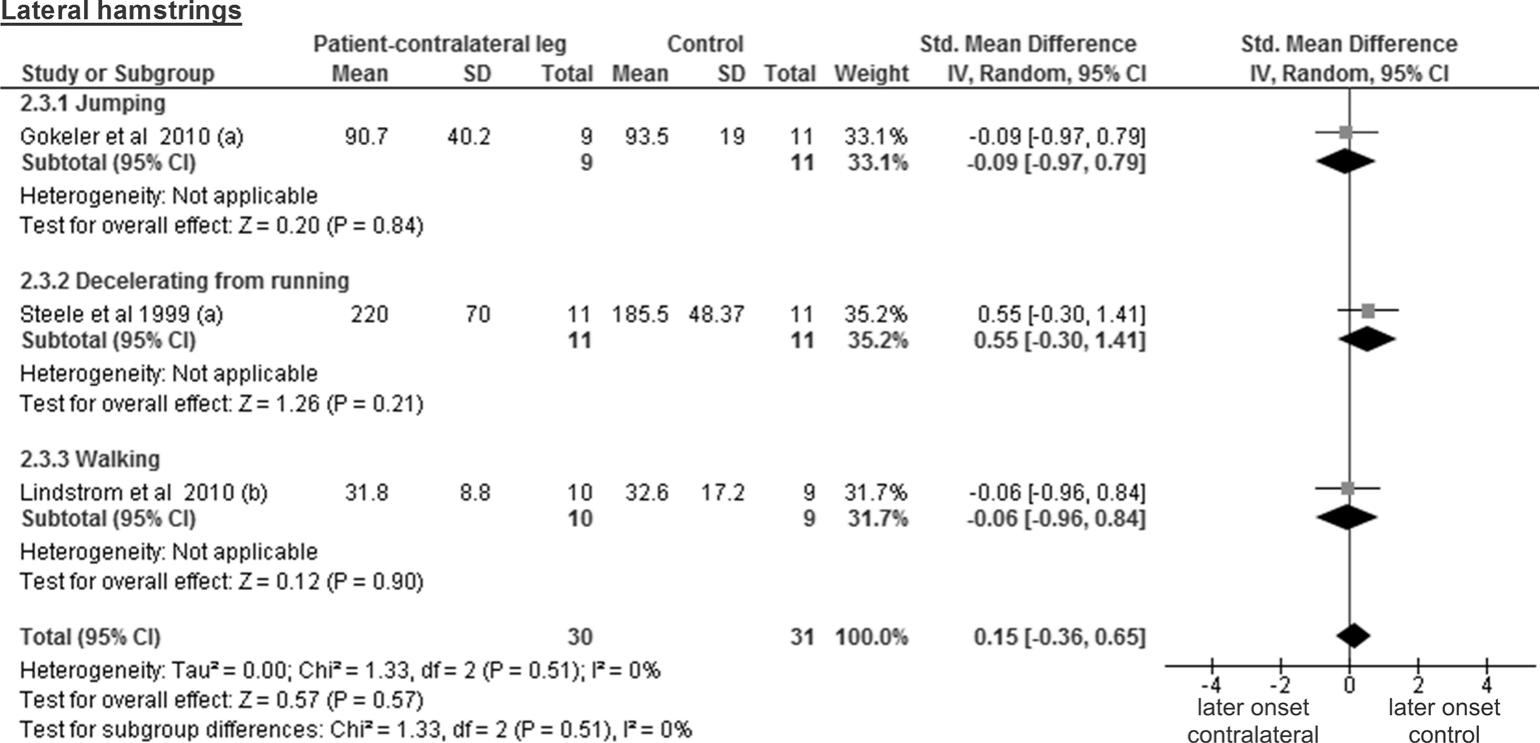
Forest plot illustrating muscle activity onset times of both groups and group differences for lateral hamstrings. SMD between ACL patients’ healthy contralateral leg (Patient-contralateral leg) and control participants (Control). (a), values in milliseconds; (b) values in percentage of stance phase.

**Fig 11:**
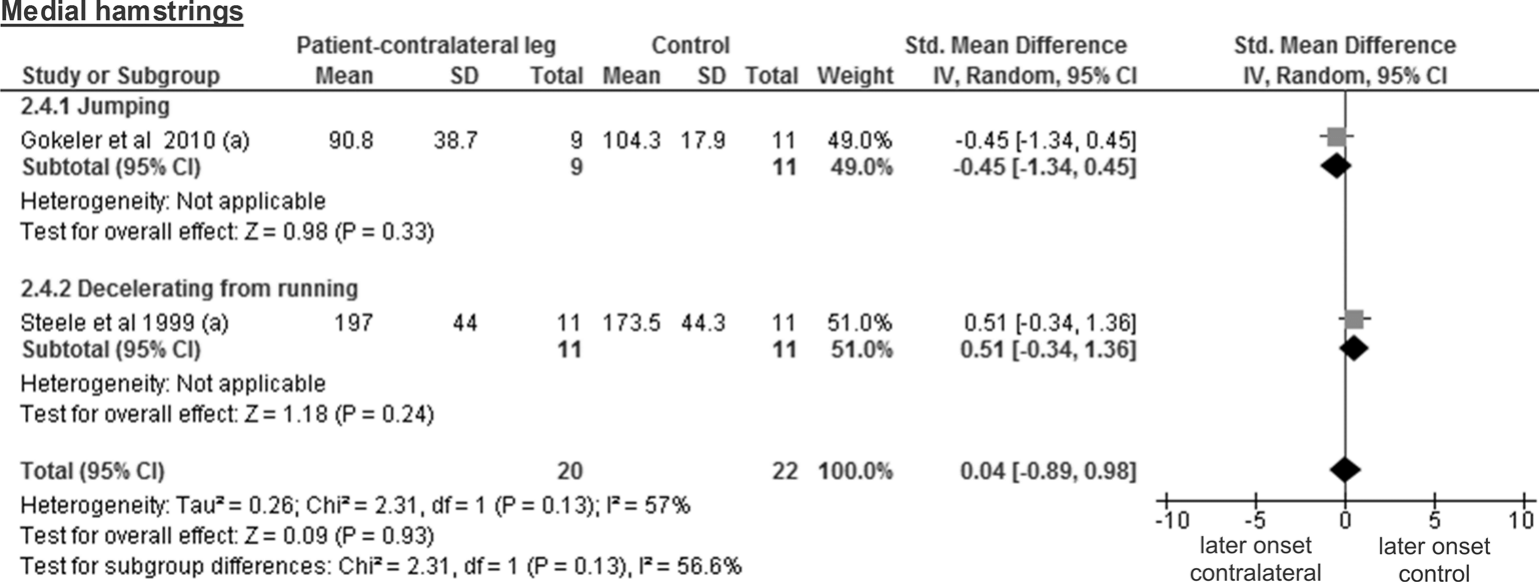
Forest plot illustrating muscle activity onset times of both groups and group differences for medial hamstrings. SMD between ACL-injured patients’ healthy contralateral leg (Patient-contralateral leg) and control participants (Control). (a), values in milliseconds.

The results for lateral hamstrings are based on three studies comparing 30 healthy contralateral legs from ACL-injured patients and 31 controls and revealed a trivial and non-significant SMD of 0.15 (95% CI -0.36 to 0.65; p = 0.57) (Fig 10). The medial hamstring findings are based on two studies including 20 healthy contralateral legs and 22 control legs. The obtained results display a trivial and non-significant SMD of 0.04 (95% CI -0.89 to 0.98; p = 0.93; moderate heterogeneity of I² = 57%) (Fig 11).

**Fig 12:**
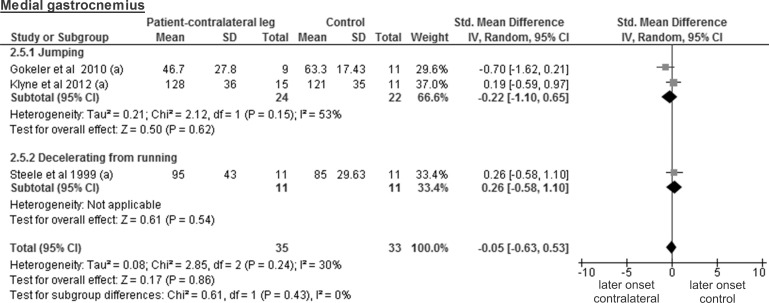
Forest plot illustrating muscle activity onset times of both groups and group differences for medial gastrocnemius. SMD between ACL patients’ healthy contralateral legs (Patient-contralateral leg) and control participants (Control). (a), values in milliseconds.

The analysis of the medial gastrocnemius included three studies with a total of 35 healthy contralateral legs and 33 control legs and revealed a trivial and non-significant SMD of -0.05 (95% CI -0.63 to 0.53; p = 0.86) (Fig 12). Again, the calculations for the lateral gastrocnemius were based on a single study concerning 9 healthy contralateral legs and 11 control legs. The SMD of -0.33 was trivial and non-significant (95% CI -1.22 to 0.56; p = 0.47).

## Discussion

The findings of the present meta-analysis, based on six eligible studies investigating muscle activity onset timing before ground contact during standardised deceleration tasks did not reveal systematic differences between ACL-injured patients and control participants. This holds true for the main knee extensor and flexor muscles of the leg concerned by the ACL injury as well as the healthy contralateral leg. Limited evidence was found for earlier onsets of muscular activity in medial gastrocnemius of ACL-patients, but this observation is based on only three studies investigating two different tasks. Furthermore, two of these studies were rated as of poor and one as of moderate quality. Weak evidence based on a single study suggests a similar phenomenon for the lateral gastrocnemius, but the quality of the study was rated poor. Although it must be acknowledged that the currently available evidence is scarce, based on the existing literature we could not conclude that ACL-injured patients have significantly different muscle activity onset timing compared to healthy control participants. It should be noted, however, that the patients included in the studies from this review were (theoretically) all rehabilitated, with times post-injury ranging from 10 months to 20 years. Obviously, more research is required to provide a definite conclusion about the role of muscle activity onset timing for optimal motor control. Furthermore, the amount of muscle activation, both prior to as well as during ground contact may be of importance, particularly in relation to “giving-way” episodes observed in some ACL-injured patients. The following paragraphs will discuss relevant factors that may have influenced our findings.

### Clinical heterogeneity

When analysing the characteristics of eligible studies, we found high clinical heterogeneity. A first aspect of heterogeneity was related to the participants recruited. Different populations of participants were represented in these studies, including both ACL-deficient and ACL-reconstructed participants. The latter group can be further classified according to the surgical technique used.

For ACL-deficient participants, the diagnosis was not established via arthroscopy in all studies, which leaves some doubt as to the status of the ACL. In prior literature a distinction was made between copers and non-copers, with the former defined as individuals who could stabilize their knee and reported no episode of giving-way during sports activities [38]. Furthermore, copers were reported to have kinetics, kinematics and muscle activation patterns similar to uninjured subjects. Conversely, non-copers conserved knee instability and had at least one episode of giving-way during their daily life activities. Also, their muscle recruitment patterns were reported to be different from those presented by healthy controls [39]. In the present review, no distinction was made between the ACL-deficient participants in terms of copers or non-copers, due to the fact that not all eligible articles provided such classification. Moreover, a variety of scales were used in the different studies to evaluate the functional ability during daily-life activities, which makes it difficult to assess if the patients recruited were homogeneous regarding their functional motor control. The ACL-deficient patients also had a highly variable time since injury, ranging from 10 months to 20 years and the injury mechanism (external impact *versus* non-contact injury) was not always specified. Regarding the post-injury rehabilitation, three studies provided no detail at all, and the question if and how muscle activation patterns change during or after rehabilitation or return to sports remains unanswered to date. The large discrepancy in participants’ post-injury status in the selected studies may hence represent a critical source of variability for the meta-analysis. Therefore these results need to be taken with some reserve.

Regarding ACL-reconstructed volunteers, 36 patients from two studies were included in the meta-analysis, 23 with a bone-patellar tendon-bone graft and thirteen with a semi-tendinosous and gracilis tendon graft [26, 27]. It has been demonstrated that the surgical technique could influence post-injury muscle recovery, potentially leading to impairments of neuro-motor control and functional performance [40, 41]. In our review, no comparison between graft types was feasible based on the limited existing data. Clearly, future investigations are required to assess the role of different graft types on muscle activity onsets during deceleration tasks during and after rehabilitation. Finally, it should be stated that the majority of the studies did not clearly report the inclusion and exclusion criteria and failed to provide detailed information about participants’ characteristics and the recruitment procedure they underwent. We are unable to confirm whether potentially confounding variables such as sex, age or level of sports practice within the recruited population could have influenced onset timing of muscular activity.

### Methodological heterogeneity

As already mentioned, four studies had an overall low quality, whereas two were classified as moderate. Full protocol descriptions associated with the correct use of the surface EMG techniques were missing in all studies. Failure to report crosstalk testing or skin preparation resulted in a negative score for item 11 of the quality check list. Additionally, some studies failed to describe the instruments used along with their sampling rates, thus scoring negatively on item 12. Due to these shortcomings in the description of EMG measurements, care is warranted regarding the interpretation of the results. Despite the use of different techniques across the included studies, the methodology to determine the onset of muscular activity was generally better described. Two studies used linear envelopes and set different thresholds for onset detection, followed by visual confirmation of their results [26, 29]. Unfortunately, these studies did not report if their experimenter was blinded when double-checking the results, which represents a risk of bias regarding the obtained onset values. One study relied solely on the visual inspection of the EMG signal by a blinded assessor [36], and one applied a mathematical algorithm termed “approximated generalized likelihood ratio” [27]. Two other techniques were also reported, one based on the detection threshold by the calculation of signal variance [28] and one relying on a multiple of the SD above the baseline EMG signal coupled with a minimum activity burst duration of 30ms [37]. None of the studies addressed the issue of reliability of their onset detection method. Given the heterogeneity in the methodologies used, and in the absence of clear guidelines, we propose that muscle activity onset detection should primarily rely on an objective technique, but should be complemented by visual verification by an assessor who is blinded regarding the muscle and the participant (patient or control) analysed. Furthermore, future studies should report on the reliability of the onset detection technique used, an important prerequisite to improve the overall confidence in the results.

In this review, the included studies investigated different deceleration tasks like running, jumping and walking. Notwithstanding the fact that they all allow analysing muscle activity onset prior to ground contact, it must be acknowledged that they are very different in terms of motor control requirements. Even for similar tasks, some degree of variability could be found between studies regarding its implementation. For example, the execution protocol of the single leg hop for distance differed between all three studies [26, 27, 36]. This may be problematic for the comparison of the results from different studies, since muscle pre-activity is likely task-specific [42]. Even changes in the conditions (e.g. jump as far as possible *versus* a set distance, walking barefoot *versus* shod, etc.) when performing the same task may modulate EMG amplitude [18]. Thus, we assume that not only the signal amplitude, but also its onset detection will be influenced by the protocol and is likely to introduce variability between the different results. Hence, before providing the reader with overall meta-analysis results, we have performed separate analyses by task for each muscle. However, close inspection of Figs 3–12 does not reveal any task-specific muscle activation pattern.

## Conclusions

Based on the results available for the present systematic review and meta-analysis, we were not able to conclude on any differences in the muscle activity onset timings between leg muscles of ACL-injured patients and healthy controls. Nevertheless, the current evidence is weak and future research should be directed towards this issue to analyse if differential muscle activity onset is associated with increased ACL injury risk, if onset timing is different in ACL injured patients and if it is modified as a result of rehabilitation. Depending on the responses to these questions, the systematic evaluation of muscle activity onset timing could be of interest in prospective screening of individuals at risk and the return to play decision for patients after an ACL injury.

## Supporting Information

S1 Table Quality checklist adapted from Munn et al 2010. (PDF)

S2 Table PRISMA checklist. (PDF)

S3 Table Quality assessment results. (PDF)
