# Supplementary material for: Muscle Activity Onset Prior to Landing in Patients after Anterior Cruciate Ligament Injury: A Systematic Review and Meta-Analysis
Source: PLoS One. 2016 May 11;11(5):e0155277. doi: 10.1371/journal.pone.0155277 (PMC4864320; doi:10.1371/journal.pone.0155277)
Supplement: S1 Table — (PDF) [file pone.0155277.s001.pdf]

**QUALITY CHECK LIST ADAPTED FROM MUNN ET AL 2010[1]**

| ITEM | Yes | unable to determine | No |
|---|---|---|---|
| ***1. Is the Hypothesis/aim/objective of the study clearly described[1]*** | ☐ | ☐ | ☐ |
| ***2. Are the main outcomes to be measured clearly described?[1]*** | ☐ | ☐ | ☐ |
| An objective method of EMG onset determination is clearly described. | | | |
| ***3. Are the characteristics of the subjects included in the study clearly described[1]*** | ☐ | ☐ | ☐ |
| The Inclusion and/or exclusion criteria and the recruitment procedure is clearly described | | | |
| ***4. Are the distributions of principle confounders in each group of subjects to be compared clearly described? [1]*** | ☐ | ☐ | ☐ |
| **Principal confounder**: Sex distribution clearly described | | | |
| Treatment Conservative vs Surgery (type) distribution clearly described | | | |
| ***5. Are the main findings of the study clearly described? [1]*** | ☐ | ☐ | ☐ |
| The author answered the question of the study so that the reader can check the major analyses and conclusions (This question does not cover statistical test which are considered below) [2] | | | |
| ***6. Does the study provide estimates of the random variability in the data for the Main outcomes? [1]*** | ☐ | ☐ | ☐ |
| In non-normally distributed data the inter-quartile range of results should be reported. In normally distributed the SE, SD or CI should be reported. If the distribution is not described it must be assumed appropriate and the answer is yes.[2] | | | |

[1] Munn J. Evidence of sensorimotor deficits in functional ankle instability: A systematic review with meta-analysis. Journal of Science and Medicine in Sport. 2010 ;( 13)1: 2–12

[2] Downs S. The feasibility of creating a checklist for the assessment of the methodological quality both of randomized and non-randomized studies of health care interventions. Epidemiol Community Health.1998; 52:377–384.

7. ***Have actual probability values been reported?***[1]

☐ ☐ ☐

Actual probability values reported (e.g.0.035) rather than <0, 05) for the main outcomes except where the probability value is less than 0.001[2]

8. ***Were the subjects participating in the study representative of the entire population from which they were recruited?***[3]

☐ ☐ ☐

The study must identify the source of ACL patients and describe how they were selected. Patients would be representative if those who agreed comprised the entire source population, an unselected sample of consecutive patients, or a random sample. Random sampling is only feasible where a list of all members of the relevant population exists. Where a study does not report the proportion of the source population from which the patients are derived, the question should be answered as unable to determine.[2]

9. ***Was an attempt made to blind those measuring the main outcomes?*** [1]

☐ ☐ ☐

The examiner of the data is blinded from the name and characteristic of the subjects. If visual inspection was used to determine the onset or performed to check the values given by an algorithm, the examiner must be blinded.

10. ***Were the statistical tests used to assess the main outcomes appropriate?***[1]

☐ ☐ ☐

Non-parametric methods should be used for small sample sizes. The parametric method is accepted when the "*data distribution appears symmetric*"[4]. Where little statistical analysis has been undertaken but where there is no evidence of bias, the question should be answered yes. If the distribution of the data (normal or not) is not described it must be assumed that the estimates used were appropriate and the question should be answered yes.[2]

---

[1] Munn J. Evidence of sensorimotor deficits in functional ankle instability: A systematic review with meta-analysis. Journal of Science and Medicine in Sport. 2010 ;( 13)1: 2–12

[2] Downs S. The feasibility of creating a checklist for the assessment of the methodological quality both of randomized and non-randomized studies of health care interventions. Epidemiol Community Health.1998; 52:377–384.

[3] The Item was modified by combining Items 11 and 12 of the Munn et at[1] quality check list

[4] Weissgerber T. Beyond Bar and Line Graphs: Time for a New Data Presentation Paradigm. PLoSBiol.2015; 13(4): 10-12

***Methodology to determine onset***

11. ***Electrode type/ placement***[5] ☐ ☐ ☐
    a) ***Electrode type***[6] :
       Is the type of electrodes clearly described (in the method section)?
    b) ***Electrode location***[6]***:*** Is the location of the electrodes on the skin clearly described (in the method section)?
    c) ***Skin preparation***[6]***:*** Was the subject's skin prepared before the placement of the electrodes (if a protocol guideline was mentioned should be answered yes)?
    *d)* ***Inter-electrodes distance***[6]***:*** Is the inter-electrode distance reported?
    *e)* ***EMG crosstalk***[6]***:*** Do the authors clearly explain if/how they tested the EMG crosstalks?

12. **Instrument used for EMG testing**[5] ☐ ☐ ☐
    *a)* ***Software/hardware***[6]***:*** Are the names of the hardware and software used specified?
    *b)* ***Sampling frequencies of data EMG***[6]***:*** Is the sampling rate of the EMG signals indicated?

*13.* **EMG Data treatment**[5] ☐ ☐ ☐
    a) ***Filter types***[6]***:*** Is the type of filter for the raw EMG signal specified? The name and characteristics (high-, low- or band-pass) of the filter should be mentioned

    *b)* ***Cut-off frequencies***[6]***:*** Are the cut-off frequencies clearly described

*14.* ***Time synchronization***[5] ☐ ☐ ☐

The methodology to determine ground contact is clearly described and the signal time is synchronized with the EMG recording.

[5]The items (10-13) were modified considering the item 20 of the Munn et al[1] check list, adapted for validity and reliability of the methodology to determine the onset time. The item has 1 only if **all** the sub items were described

[6] Standards for Reporting EMG Data. Journal of Electromyography and Kinesiology. 2015. 25:1-2.

***15. Were the controls matched with the ACL patients?*** [7]

☐ ☐ ☐

The control group was matched with the ACL patients for: age, sex distribution, activity level with no significance difference between groups

***16. Was there adequate adjustment for confounding in the analyses from which the main findings were drawn?*** [8]

☐ ☐ ☐

Are separate results presented for subgroups if the groups are not homogeneous regarding age, sex distribution or activity level, or if the patient group comprises participants having had surgery and patients with conservative treatment?

[7]The item is was modified from the original item 21 from Munn[1] et al check list
[8]The item was modified from the original item 25 from Munn[1] et al check list
