# Supplementary material for: Muscle Activity Onset Prior to Landing in Patients after Anterior Cruciate Ligament Injury: A Systematic Review and Meta-Analysis
Source: PLoS One. 2016 May 11;11(5):e0155277. doi: 10.1371/journal.pone.0155277 (PMC4864320; doi:10.1371/journal.pone.0155277)
Supplement: S3 Table — (PDF) [file pone.0155277.s003.pdf]

	**Bryant et al. 2009**	**Gokeler et al. 2010**	**Klyne et al. 2012**	**Steele et al. 1999**	**Lindstrom et al. 2010**	**Lass et al. 1991**
Hypothesis/objective clear	YES	NO	YES	NO	NO	NO
Main outcomes clearly described	YES	YES	YES	YES	YES	YES
Characteristics of subjects fully described	NO	NO	YES	NO	NO	NO
Distribution of principle confounders described	YES	YES	YES	NO	YES	NO
Main findings clearly described	YES	NO	YES	YES	NO	NO
Provide estimates of random variability	YES	YES	YES	YES	YES	YES
Probability values reported for main outcomes	YES	YES	YES	YES	YES	NO
Evidence of participants representative of the population recruited	NO	NO	YES	NO	NO	NO
Blind measuring of the main outcomes reported	NO	YES	YES	NO	YES	YES
Appropriate statistical test to assess outcomes	YES	YES	YES	YES	YES	NO
Electrode type/placement protocol reported	NO	NO	NO	NO	NO	NO
Instrument used for EMG testing described	YES	YES	YES	NO	NO	NO
EMG data treatment clearly described	YES	YES	YES	YES	NO	YES
Time synchronization described	YES	YES	NO	NO	YES	YES
Controls matched with ACL patients and its statistical evidence	NO	NO	NO	NO	NO	NO
Adequate adjustment for confounding in the analysis	YES	NO	YES	NO	NO	NO
**Total % score**	61%	50%	72%	33%	39%	28%
**Overall Quality**	Moderate	Low	moderate	Low	Low	Low
